# Blood levels of copper, manganese, selenium, and zinc are positively associated with cognitive function and academic performance in adolescents

**DOI:** 10.3389/fnut.2025.1638283

**Published:** 2025-07-24

**Authors:** Abdur Rahman, Muddanna Rao, Ahmed Aldughpassi, Reem Jallad, Lemia Shaban

**Affiliations:** ^1^Department of Food Science and Nutrition, College of Life Sciences, Kuwait University, Kuwait City, Kuwait; ^2^Department of Anatomy, Faculty of Medicine, Kuwait University, Kuwait City, Kuwait

**Keywords:** microminerals, cognition, academic performance, adolescents, school children

## Abstract

**Background:**

Microminerals copper (Cu), manganese (Mn), selenium (Se), and zinc (Zn) regulate neuronal signaling and brain function. Deficiencies of these minerals are common in adolescents due to unhealthy eating habits. We investigated the association between micromineral levels and cognitive function and academic performance in a nationally representative sample of adolescents.

**Methods:**

Adolescents (*N* = 1,370; 11–14 years) were selected from public middle schools in Kuwait, using cluster random sampling. Data on various covariates were collected through a questionnaire from the subjects and their parents. Blood micromineral levels were determined using Inductively Coupled Plasma Mass Spectrometry (ICP-MS). Cognitive function was evaluated through the Raven’s Standard Progressive Matrices test and academic performance data were taken from the school records.

**Results:**

Median (IQR) blood levels of microminerals (μg/dL) were as follows: Cu, 22.5 (15.2, 33.0); Mn, 6.0 (3.5, 10.6); Se, 34.2 (22.1, 52.5); Zn, 163.5 (118.5, 233.5). On average, SPM score was higher by 5 points in adolescents with mineral levels above the median, compared to those with levels below median (*p* < 0.05). Academic performance in adolescents with micromineral levels in Q1 was lower by an average of seven-percentage points compared to those in Q4 (*p* < 0.01). Multivariable regression analysis showed positive association (*p* < 0.01) with both cognitive function and academic performance, whether the micromineral levels were used as continuous variable or as quartiles.

**Conclusion:**

In this cohort, blood micromineral levels are positively associated with cognitive function and academic performance. Improving micromineral status by public health intervention is strongly warranted, as academic performance during early-life education has implications on the later-life socio-economic status.

## Highlights

- What is already known on this topic: Microminerals play key roles in brain development and function, but adolescents generally do not meet micromineral requirements due to poor dietary habits.
- What this study adds: As data from properly designed studies on the association between micromineral levels and cognition and academic performance is limited, this study reports a positive association between blood levels of microminerals (copper, manganese, selenium, and zinc) and cognition and academic performance based on a nationally representative sample of adolescents.
- How this study might affect research, practice or policy: Early-life education has life-long socio-economic implications. This study calls for correcting micromineral status during adolescence by public health interventions.

## Introduction

Nutrition plays a critical role in the development and functioning of the brain during the prenatal period and infancy (1), childhood and adolescence (2) and adulthood (3). Microminerals (also known as trace elements) include iron (Fe), iodine (I), zinc (Zn), copper (Cu), selenium (Se), and manganese (Mn). Of these, Zn is the second most abundant micromineral and is found in almost every tissue (4), and is involved in several metabolic functions including brain development and cognitive function (5, 6). Se is another essential micromineral which functions as an antioxidant and protects cellular membranes from oxidative stress. It is a major component of several functional proteins and enzymes, most importantly, the selenoproteins which are associated with thyroid hormones activation and brain functions (7, 8). Cu and Mn are also essential trace elements in the human body. Both function as enzymes cofactors, regulating normal metabolic functions (9). Both Cu and Mn are redox-active minerals and thus can work as antioxidants providing defense against oxidative stress and free radical damage. Both low and high Cu and Mn levels are known to negatively affect the IQ and cognitive functions in humans (10–12).

Deficiencies of microminerals are fairly common, and low levels of these minerals have been shown to adversely affect cognition and psychological behaviors (13). Micronutrient deficiencies in general are common in the Middle East, particularly in children, due to high consumption of fast foods and other unhealthy eating patterns. In Kuwait, poor nutrient intake and obesity among children are highly prevalent. However, to our knowledge, no data are available on the association of blood micromineral levels with cognitive function and academic performance in adolescents in Kuwait. We hypothesized a positive association between blood levels of microminerals (Cu, Mn, Se, and Zn) with cognitive function and academic performance in school in Kuwait. This hypothesis was tested in a nationally representative sample of adolescents.

## Materials and methods

### Study design and protocol

This cross-sectional study was based on subjects selected from public middle schools from all the six governorates in Kuwait. Ethical approval for the study was obtained from the Institutional Ethics Committee of Kuwait University (reference No. DR/EC/2338; dated 2/6/2015) and the Ministry of Health, Kuwait (2015/248; and the amended version 2022/2072). The study was conducted in accordance with the Declaration of Helsinki ethical principles for medical research involving human subjects. Written informed consent of the parents/guardian and verbal assent of the participants were obtained. Subjects were selected using a multistage cluster random sampling method with probability proportionate to size of each Governorate. Data on various covariates were collected from the parents through a self-administered questionnaire and from the adolescents using face-to-face interviews. Details of the subject selection, including exclusion and inclusion criteria, and sample size calculations have been previously published (14, 15).

### Cognitive function tests

Cognitive function was tested using the Raven’s Standard Progressive test (SPM), as described in Rahman et al. (15). We used the Arabic version of this test, which has been adapted and validated by the Ministry of Education for use in Kuwait. The test is designed to measure the observation and thinking capacities of the individuals and is based on analyzing the relationship between meaningless figures through a systematic method of reasoning. SPM is an index of a person’s intellectual capacity irrespective of the person’s culture, language, education, ethnicity and physical condition. Raw scores were converted into the standard and percentile scores as per the instruction manual (2008, Pearson Education Inc., London, UK).

### Academic performance

Data on the academic performance was extracted from the schools’ records. We obtained the score of each participant (as percentage) in mathematics, science and English in an exam conducted closest to the date of blood collection. We also recorded the overall percentage score that the students obtained in all subjects put together (Total Score). The total score included other study subjects such as physical education and Arabic literature.

### Blood collection and micromineral analyses

After obtaining written informed consent of the parents and verbal assent of the students, a trained pediatric nurse collected 5 mL of venous blood from each subject. Blood samples were analyzed in a tertiary care hospital for CBC and various biochemical parameters, including glucose, iron profile, calcium, PTH and serum 25-hydroxyvitamin D. For mineral analyses, whole blood (0.5 mL) was digested in 5 mL of perchloric acid/nitric acid (1:5) mixture. After complete digestion of the samples, the perchloric acid/nitric acid solution was evaporated and the residue was dissolved in 5 mL of 1% nitric acid. To avoid contamination, all glassware used were washed with nitric acid, and double-deionized water was used in all sample preparation steps. Mineral analysis was carried out by Inductively Coupled Plasma Mass Spectrometry (ICP-MS). Lyophilized whole -blood samples of known mineral content (Clin-Check- Control, Cat. #884042; Recipe, Munich, Germany) were used for quality control checks.

### Statistical analysis

The distribution of predictors (micromineral levels) and the outcome variables (SPM) score and school scores was evaluated using histograms and normal plots. As the data were not normally distributed and could not be normalized with transformation, data are expressed as median and interquartile range (IQR). The association between each micronutrient and cognitive function was assessed by univariable and multivariable linear regression, using the micromineral data as continuous variables as well as quartiles. Coefficients were calculated in both unadjusted as well as adjusted multivariable analysis. In the adjusted models, all the variables which were significantly associated with cognitive function (15) in this cohort were entered into the final model. Median test for independent samples was used to compare the school scores and RPM scores across various quartiles of microminerals. SPSS version 26 was used for analyzing the data. A *p*-value of <0.05 was used for significance.

## Results

**Figure 1:**
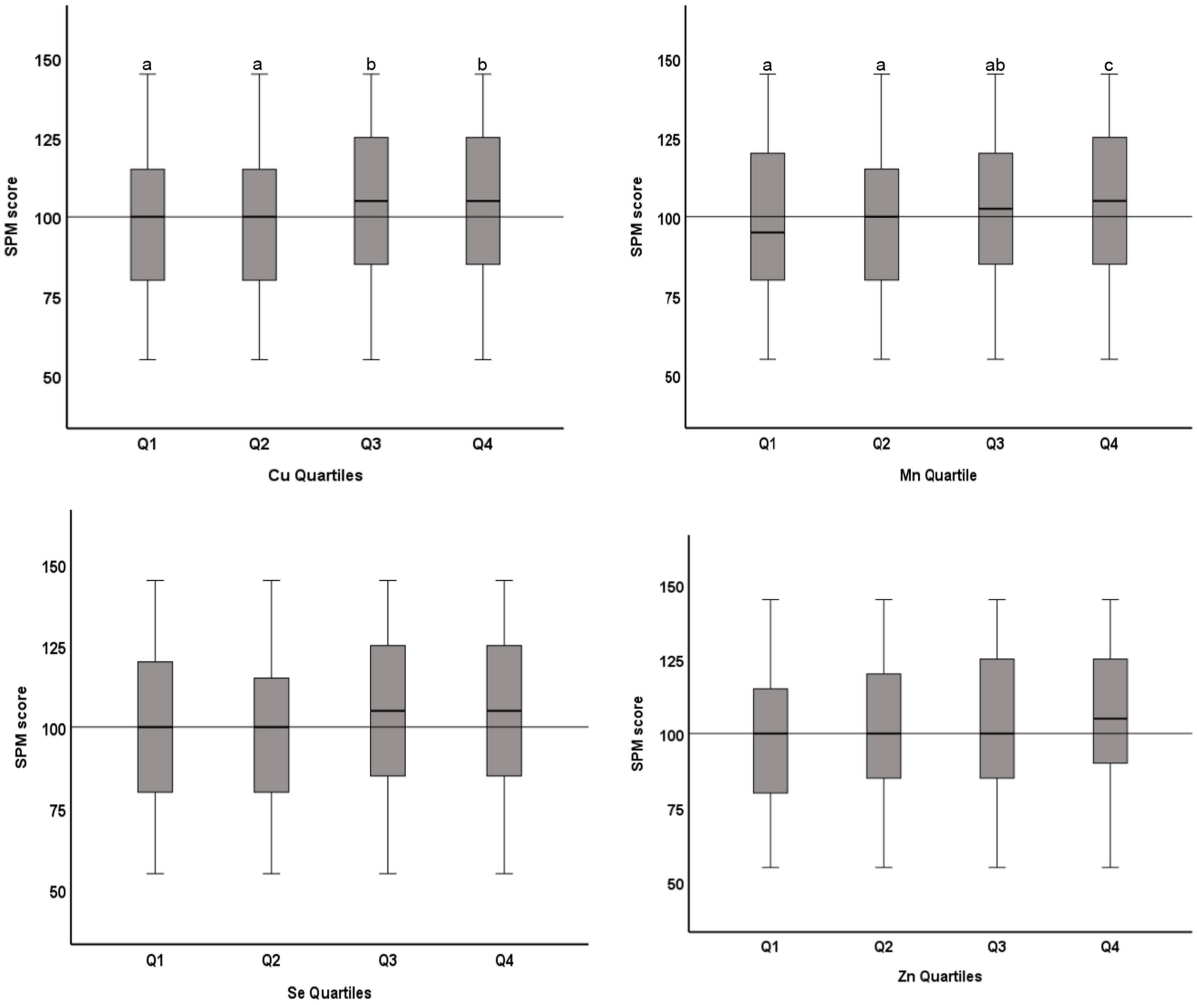
Distribution of Raven’s SPM score across quartiles of microminerals: Boxplot showing distribution of Raven’s Standard Progressive Matrices (SPM) score across the quartiles of Cu, Mn, Se, and Zn. Boxes show the distribution of SPM score as median and interquartile range. Data were analyzed by independent sample Kurskal-Wallis test; Cu Quartiles (*p* = 0.04); Mn Quartiles (*p* < 0.001); Se Quartiles (*p* = 0.08); Zn Quartiles (*p* = 0.06). Boxes marked by different superscript letters are statistically different (*p* < 0.05). The horizontal line represents the overall median (100).

**Figure 2:**
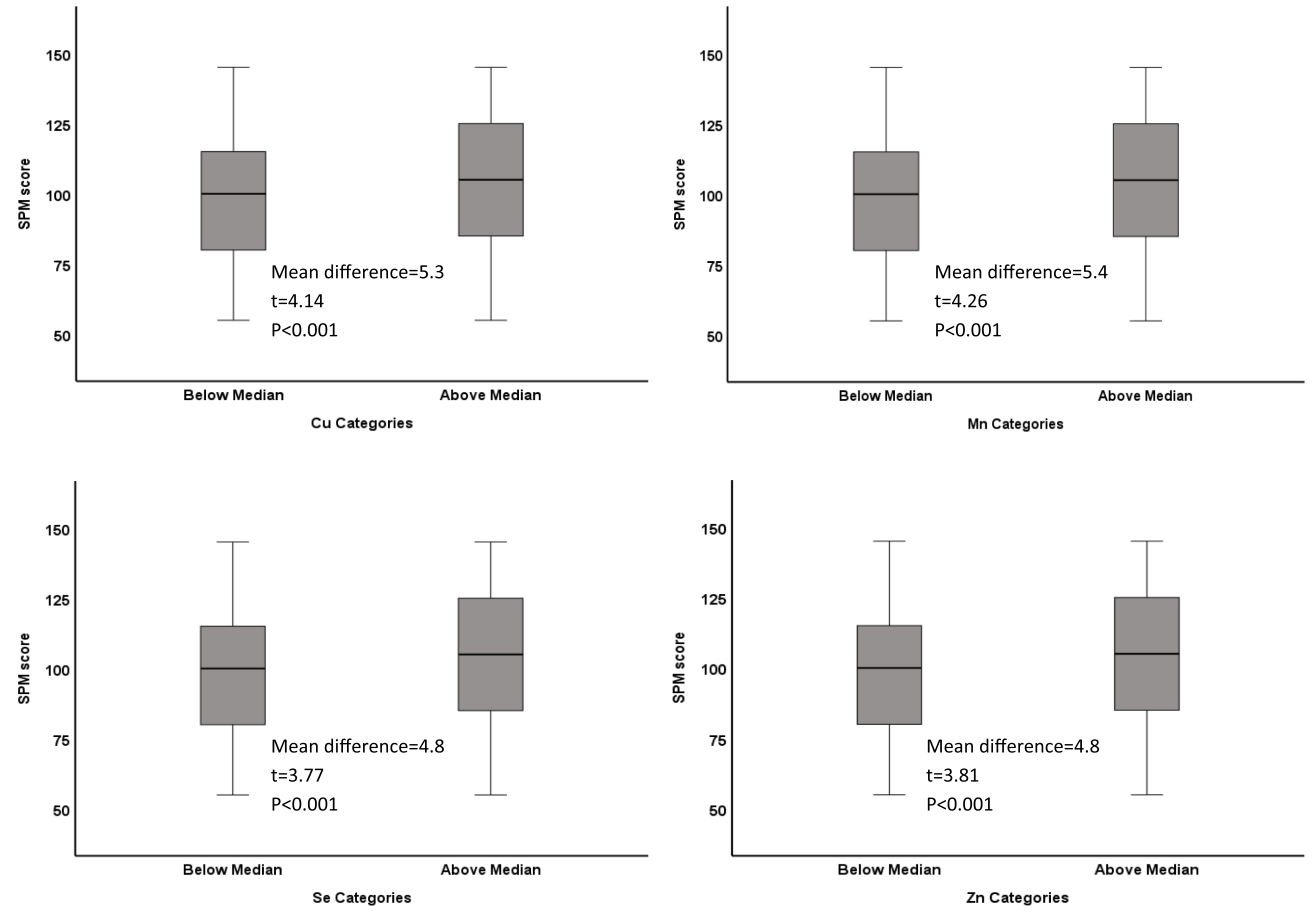
Distribution of Raven’s SPM score in low and high categories of microminerals: Boxplot showing distribution of Raven’s Standard Progressive Matrices (SPM) score by categories (below median and above median) of Cu, Mn, Se, and Zn. Boxes show the distribution of SPM score as median and interquartile range. Data was analyzed by t-test for independent samples. The mean difference between the two categories, along with their respective t-statistic and the *p*-value for each mineral is shown on each plot.

Of the 1,422 students who returned the consent forms, blood samples were collected from 1,416 students. Micromineral data were available for 1,396 students, of which 681 were boys and 715 were girls. Mean (SD) age was 12.48 (0.93) years, ranging from 11 to 14 years. Socio-demographic characteristics of the study population are previously published (14, 15). Median (IQR) blood levels of microminerals (μg/dL) are shown in Table 1. Median Cu and Se levels in boys were significantly higher than girls (*p* < 0.05), whereas Mn and Zn levels were not significantly different between boys and girls. Figure 1 shows the distribution of SPM scores over the quartiles (Q1–Q4) of the microminerals. As shown, SPM scores were significantly higher in Q4 compared to the lower quartiles of Cu and Mn (*p* < 0.05). The difference in the SPM score across the quartiles of Se and Zn were marginally significant (*p* = 0.08 for Se and 0.06 for Zn). When the SPM scores of children with micromineral levels below the median were compared with those who had micromineral levels above the median, the mean difference between the groups (in favor of the higher group) was 5.3, 5.4, 4.8, and 4.8 points (*p* < 0.001 for each micromineral) for Cu, Mn, Se, and Zn, respectively (Figure 2). Table 2 shows the association between the microminerals and the SPM scores. As shown, all the microminerals were positively associated (*p*-values for all minerals <0.01) with the SPM score, whether the micromineral levels were used as continuous variable (Model 1) or as quartiles (Model 2). This positive association remained significant when the models were adjusted for confounding variables (Models 3 and 4, respectively). It is of interest to note that although the median differences across the quartiles of Se and Zn were marginally significant (Figure 1), after adjusting for other confounding variables (Model 4), the associations became highly significant. The coefficients (*β*) in the final adjusted model, when microminerals were used as quartiles (Model 4), were 2.03, 2.47, 1.64, and 1.88, respectively for Cu, Mn, Se, and Zn (*p* < 0.01 for all).

**Table 1:** Median (IQR) blood micromineral concentrations (μg/dL) in adolescents.

		Whole blood microminerals (µg/dL)			
		Cu	Mn	Se	Zn
Total	Median	22.5	6.0	34.21	164
	IQR	15.2, 33.0	3.5, 10.6	22.12, 52.49	119, 234
Boys	Median	24.0*	5.7	37.7*	167
	IQR	15.8, 32.4	3.1, 11.2	22.4, 52.2	122, 222
Girls	Median	21.3	6.4	31.1	160
	IQR	14.9, 34.4	3.7, 10.0	21.6, 52.9	115, 253

*Significantly different from girls by median test for independent samples (*p* < 0.05). IQR, inter-quartile range.

**Table 2:** Regression analyses showing association between microminerals and SPM score in adolescents.

		Whole blood microminerals			
Models	Regression parameters	Cu	Mn	Se	Zn
Model 1	Coefficient (*β*)	0.15	0.16	0.07	0.03
	95% CI	0.06–0.25	0.07–0.26	0.02–0.13	0.02–0.05
	*p*-value	0.002	0.001	0.008	<0.001
Model 2	Coefficient (*β*)	2.18	2.69	1.89	2.61
	95% CI	1.07–3.29	1.58–3.80	0.78–3.00	1.50–3.72
	*p*-value	<0.001	<0.001	0.001	<0.001
Model 3	Coefficient (*β*)	0.13	0.12	0.05	0.03
	95% CI	0.04–0.22	0.02–0.21	−0.01-0.10	0.01–0.04
	*p*-value	0.007	0.019	0.083	0.001
Model 4	Coefficient (*β*)	2.03	2.47	1.64	1.88
	95% CI	0.92–3.13	1.36–3.57	0.54–2.74	0.79–2.97
	*p*-value	<0.001	<0.001	0.003	0.001

CI, confidence interval.

Model 1: unadjusted; independent variable used as continuous variable.

Model 2: unadjusted; independent variable used as quartiles.

Model 3: independent variable used as continuous variable and model adjusted for sex, smoking in the household, school grade, father education, mother education, season of birth, soft drinks.

Model 4: independent variable used as quartiles and model adjusted for as in model 3.

**Figure 3:**
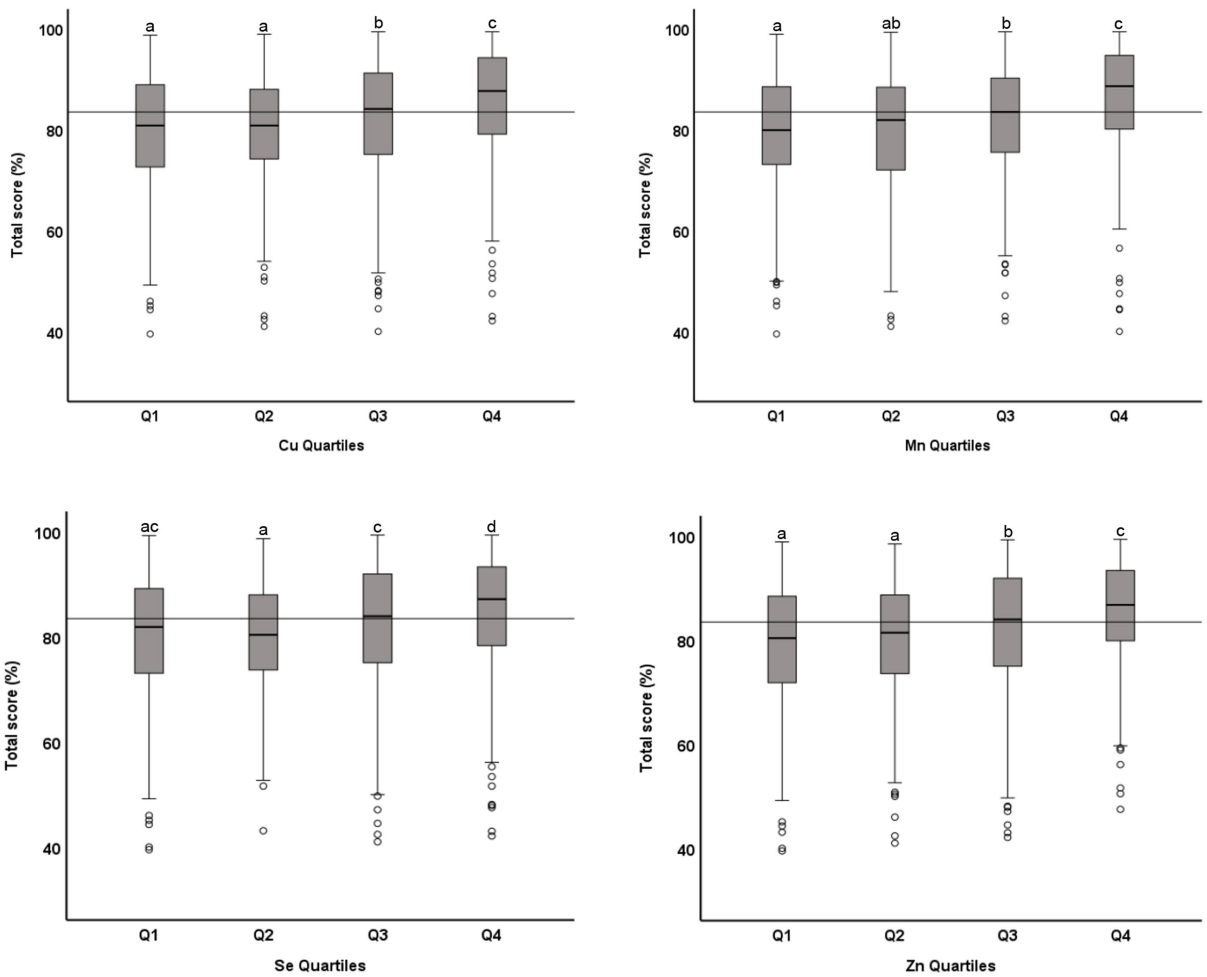
Distribution of academic performance (toral percentage score) across quartiles of microminerals: Boxplot showing distribution of total percentage score, obtained in one exam taken closest to the blood draw, across the quartiles of Cu, Mn, Se, and Zn. Data were analyzed by independent sample median test; Boxes marked by different superscript letters are statistically different (*p* < 0.05). The horizontal line represents the overall median (83.5).

**Figure 4:**
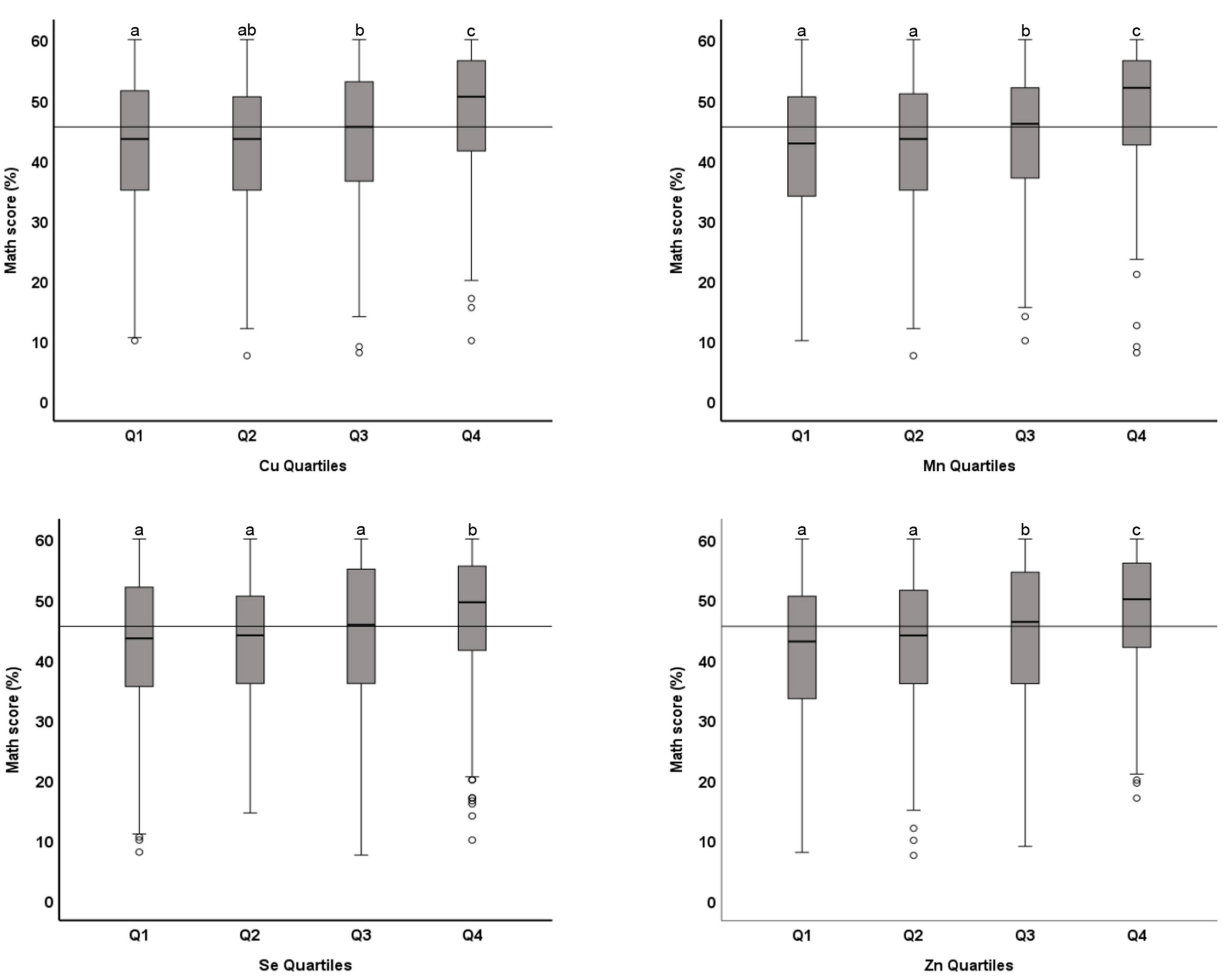
Distribution of academic performance (percent score in mathematics) across quartiles of microminerals: Boxplot showing distribution of percentage score in mathematics, obtained in one exam taken closest to the blood draw, across the quartiles of Cu, Mn, Se, and Zn. Data were analyzed by independent sample median test; Boxes marked by different superscript letters are statistically different (*p* < 0.05). The horizontal line represents the overall median (45.5).

The association between microminerals and academic performance in mathematics is shown in Table 3. All micromineral tested (Cu, Mn, Se, and Zn) showed positive association (*p* < 0.001) with the percentage score in mathematics, whether the microminerals levels were used as continuous variable (Model 1) or as quartiles (Model 2). This positive association remained significant when the regression models were adjusted for confounding variables (models 3 and 5). We also tested the association between micromineral levels and academic performance in science and English and in the total score and got similar results (data not shown). Figure 3 shows the distribution of the total score across the quartiles of microminerals. While there was no statistically significant difference between Q1 and Q2, the higher quartiles (Q3 and Q4) were significantly different from the lower quartiles (*p* < 0.05). Similarly, the distribution of the percentage score in mathematics across the quartiles of microminerals is shown in Figure 4. The results are largely similar to the total score. On average, there was a seven-percentage point difference in the mathematics score between the Q1 and Q4 of the microminerals.

**Table 3:** Regression analyses showing association between microminerals and academic performance (mathematics) in adolescents.

		Whole blood microminerals			
Models	Regression parameters	Cu	Mn	Se	Zn
Model 1	Coefficient (*β*)	0.15	0.13	0.07	0.03
	95% CI of *β*	0.11, 0.20	0.09, 0.18	0.04, 0.09	0.02, 0.03
	*p*-value	<0.001	<0.001	<0.001	<0.001
Model 2	Coefficient (*β*)	1.70	2.27	1.40	1.96
	95% CI of *β*	1.20, 2.20	1.76, 2.78	0.91, 1.95	1.45, 2.48
	*p*-value	<0.001	<0.001	<0.001	<0.001
Model 3	Coefficient (*β*)	0.12	0.10	0.05	0.02
	95% CI of *β*	0.08, 0.16	0.05, 0.14	0.03, 0.08	0.01, 0.03
	*p*-value	<0.001	<0.001	<0.001	<0.001
Model 4	Coefficient (*β*)	1.40	1.60	1.00	1.50
	95% CI of *β*	0.90, 1.90	1.10, 2.10	0.60, 1.50	1.00, 2.00
	*p*-value	<0.001	<0.001	<0.001	<0.001

CI, confidence interval.

Model 1: unadjusted; independent variable used as continuous variable.

Model 2: unadjusted; independent variable used as quartiles.

Model 3: independent variable used as continuous variable and model adjusted for sex, father education, mother education (which were positively associated with academic performance) and for someone smoking in the household and household income (which were negatively associated with academic performance).

Model 4: independent variable used as quartiles and model adjusted for as in model 3.

## Discussion

The data presented in this study demonstrates a strong positive association between the microminerals blood levels and cognitive function, as assessed by Raven’s Progressive Matrices, and academic performance, both in individual subjects like science and mathematics and the composite score in all subjects. On average, a seven-percentage point difference between the lower quartile (Q1) and the highest quartile (Q4) of the various microminerals tested was observed. These findings have significant public health implications, as micromineral deficiencies have been widely reported as a public health problem in various parts of the world. While this is a cross-sectional study from which a cause-and-effect relationship cannot be established, mechanistic data from the literature about microminerals and cognition make the positive association between the studied microminerals and cognitive function relevant. In the subsequent sections, we discuss the nutritional status of these minerals and the potential mechanisms by which these minerals affect cognition.

### Zn and cognition

Zn is the predominant trace mineral in the CNS. The average Zn concentration in the brain is 10 mg/g (wet weight), which is higher than any other tissue (16). Zn plays an essential role in the CNS across the lifespan, starting from neonatal brain development through adulthood (17). Zn is essential for the development of the CNS by affecting stem cell formation, cell proliferation, and differentiation during neurodevelopment (17, 18). Zn affects CNS development and function in several ways. These include (1) affecting neurotransmitters production, function, and release, (2) affecting the expression and function of brain metalloenzyme and proteins, and (3) affecting the transcription of various genes through Zn fingers. It also interacts with critical hormones and other cofactors that are associated with brain functions, cognition, memory, and information processing (19, 20). Zn is abundantly present in neurons in several brain regions, including the dentate gyrus, hippocampus, piriform cortex and amygdala (21). The high concentration of Zn in the synaptic vesicles of neurons in several brain areas clearly suggests a role for Zn in synaptic functions (22). Zn is released at glutamatergic synapses in response to depolarization and acts through the NMDA and GABA receptors (21, 22). Based on its diverse functions in the CNS, it is expected that the Zn deficiency would be associated with poor cognition, especially during the CNS growth and development such as in infancy and adolescence (23, 24). Low to severe Zn deficiency was reported in children with Autism, attention deficit hyperactivity disease, Schizophrenia, Alzheimer’s disease, and depression (21, 23). The positive association between Zn and cognition that we observed in this study is supported by several studies that have reported low cognitive function in Zn deficiency (epidemiological) and improvement in various aspects of cognition with Zn supplementation (experimental) in children and adolescents (25–28).

### Cu and cognition

The human brain contains approximately 4–5 mg/g wet weight Cu, mostly in the gray matter (29, 30). Cu-containing enzymes in the brain are involved in neurogenesis and neurotransmission (12, 31, 32). A defect in Cu balance alters these enzymes’ functions and affects oxidative defense, energy production, myelination, hormone production, neurogenesis, and overall CNS metabolism, with adverse neurological manifestations (31, 33, 34). The two most common Cu containing enzymes in the brain are dopamine-β monoxygenase (DβM) and peptidyglycine α-amidating monoxygenase (PAM) (12). DβM catalyzes the final step in converting tyrosine to norepinephrine (35). PAM is the only enzyme that catalyzes the α-amidation of peptide precursors which are involved in a variety of brain functions including neuronal proliferation, energy metabolism and neuromodulation (36). Cu is found in synaptic vesicles and cleft, highlighting its role in membrane function and structure (32). However, studies on the association of Cu status with cognitive functions are scarce. Cu deficiency is associated with Alzheimer’s disease and other neurodegenerative diseases with cognitive impairment (37). On the other hand, some studies have shown an association between elevated Cu levels and low cognition in children and adults (38, 39).

### Mn and cognition

The amount of Mn in nervous tissue ranges between 1 and 2 μg/g dry weight (40). Mn can cross the blood–brain barrier (BBB) and blood-cerebrospinal fluid barrier (BCB) through several transporters and in different oxidation states in a tightly controlled manner (41, 42). The most important enzymes related to Mn in the brain is glutamine synthetase, which accounts for 80% of brain Mn (43). Glutamine synthetase regulates the levels of GABA, glutamate and glutamine at the synapse and thus synaptic excitation or inhibition (44). The CNS produces a high amount of ROS, which requires antioxidants for the protection of the brain cells and function. Mn dependent superoxide dismutase (MnSOD) is a mitochondrial enzyme which protects the brain from oxidative damage. MnSOD is mostly found in cerebral neurons and to a lesser extent in glial cells (41). The role of these enzymes in the brain function and integrity clearly suggests a role for Mn in the CNS. However, the data on the association between blood levels of Mn and cognition in children and adolescents are scarce. Our data presented here will add to the limited data available on this subject.

### Se and cognition

The human brain contains 90–110 ng/mg wet weight of Se (45). Although, this level is lower than in other tissues like kidney and liver, brain preferentially retain Se in conditions of deficiency (46). Selenoproteins are abundantly expressed in the brain (47). The brain has a high level of polyunsaturated fatty acids, high oxygen demand, neurotransmitter auto-oxidation, but a modest level of antioxidants. As such, it is susceptible to high oxidative stress (48). Selenoproteins function as neuroprotective antioxidants in the brain, particularly SeP and GPx (49, 50), and inborn errors of SeP synthesis accelerate neurodegeneration and alter neuronal development (51). The role of thyroid hormones in brain development and cognition is well established. Se plays a functional role in thyroid hormone synthesis, activation, and protection of the thyroid glands against oxidative damage (52, 53). Thus, Se may influence cognitive performance, mood, and behavior through thyroid hormones disturbance. In several studies, Se deficiency was associated with poor cognitive performance in infants, school children and elderly (54–58). Low dietary intake of Se has also been linked to psychiatric disorders in adult women (59). Se was found to be low in some neurological disease such as Parkinson’s disease, epilepsy, dementia, and Alzheimer’s disease, linking it with the etiology or the manifestation of these conditions. However, further studies are needed to confirm the true association between Se and the incidence of these diseases (60, 61).

## Micromineral status

Zn deficiency is a public health problem worldwide (62). According to the world health organization (WHO), Food and Agriculture Organization (FAO) and International Zinc Nutrition Consultative Group (IZiNCG), 20% of the world population is at risk of developing Zn deficiency. An estimated 15–20% of the world population does not meet their RDA for Zn (63). Zn deficiency has been reported from many countries, in children, adolescents, and women of reproductive age (27, 64–69). Cu deficiency (based on plasma/serum Cu levels) has also been reported from several countries (70–72).

Limited data are available on the Cu and Zn status of Kuwaiti population. An earlier study in Kuwait on a relatively small sample size and on a rather broader age range (15–80 years old) reported that 36% of the general population is Cu deficient, whereas Zn deficiency was reported to be of no significant public health importance (73). Although the current study is based on a nationally representative sample of adolescents in Kuwait, we are unable to report the estimates of micromineral deficiencies in this population due to the absence of local references ranges for the whole blood micromineral levels.

The absence of established age-specific local cutoffs makes it difficult to determine the prevalence of deficiencies of these microminerals in adolescent population in Kuwait. This is further complicated by the variations in the specimen (whether plasma, serum or whole blood) used for analysis. While the values for some minerals are quite similar in serum/plasm and whole blood, it is significantly different for others. For example, Cu levels of serum and whole blood were reported to be quite similar, whereas serum Zn levels were significantly lower than whole blood Zn levels (74–76). Despite these issues, our data reveals that Cu and Zn levels in our population are significantly lower than the blood levels of these micromineral reported in the literature across various age groups from various countries (Supplementary Table 1). Median Cu level in our population was 23 μg/dL compared to the reported means (median or GM) ranging from 87 to 124 μg/dL. Similarly, median Zn level in population was 164 μg/dL, compared to the reported means (median or GM) ranging from 470 to 760 μg/dL. On the other hand, blood levels of Mn and Se in our study population were higher than all the studies reported (Supplementary Table 1). Adolescence growth spurt which increases the requirements for these minerals due to rapid growth, inadequate dietary intake due to overall poor dietary habits, higher levels of toxic heavy metals like Pb (14); and cadmium (unpublished data from this cohort) may increase the prevalence of these micromineral deficiencies.

## Strengths and limitations

This is the first systematically conducted study on a nationally representative sample that investigated the association between micromineral levels and cognitive function and academic performance. Another strength of this study is that we tested cognitive function using a language and culture-free test (Raven’s SPM) to minimize the influence of language and culture. SPM is the most widely used non-verbal test of cognition in subjects with a wide age range. Furthermore, we gathered data on academic performance on several subjects individually, like science, mathematics and English, as well as the composite academic score. We used a variety of statistical tools that show the robustness of the results. In addition, the regression models were adjusted for all the confounding factors that were associated with cognitive function and academic performance in this cohort. In addition, we employed the most sensitive method of mineral estimation (ICP-MS) with strict quality control measures. A limitation of this study is that a single measure of cognition (Raven’s SPM) was used which does not measure all cognitive abilities. In addition, dietary history and food intake were not evaluated as possible factors for micromineral status. The overall nutritional status (and dietary intake) is a known predictor of cognition, and it could be argued that the blood micromineral levels reflect the overall poor diet, which could have confounded the reported association between micromineral levels and cognitive function and academic performance. This hypothesis, however, does not seem plausible for two reasons. One, only a small proportion of the adolescents (<2%) were underweight, the remaining population was either normal, weight, overweight or obese. As such overall undernutrition is less likely to be a confounding factor. Second, we tested the association of several blood parameters that reflect nutritional status (including Fe, hemoglobin, vitamin B_12_, folate and vitamin D) and none of these were associated with SPM score or academic performance.

In conclusion, this study highlights a very strong statistically significant positive association between micromineral levels and cognitive function and academic performance. Low cognitive function and academic performance during school age has life-long socio-economic implications. These findings have significant public health implications, as microminerals levels in adolescents are generally considered to be marginal. Further research is warranted to develop local age-specific cutoffs and to estimate the true prevalence of these micromineral deficiencies in the population.

## Data Availability

The datasets presented in this article are not readily available because the project has been converted into a longitudinal cohort study. Requests to access the datasets should be directed to the corresponding author, AR.

## References

[1] Nyaradi A Li J Hickling S Foster J Oddy WH. The role of nutrition in children's neurocognitive development, from pregnancy through childhood. Front Hum Neurosci. (2013) 7:97. doi: 10.3389/fnhum.2013.00097, PMID: 23532379 PMC3607807

[2] Lifshitz F Tarim O Smith MM. Nutrition in adolescence. Endocrinol Metab Clin N Am. (1993) 22:673–83. doi: 10.1016/S0889-8529(18)30157-9, PMID: 8243454

[3] Maurage C. Children's nutrition and health in adulthood. Appetite. (2008) 51:22–4. doi: 10.1016/j.appet.2008.02.005, PMID: 18395932

[4] Pfeiffer CC Braverman ER. Zinc, the brain and behavior. Biol Psychiatry. (1982) 17:513–32. 7082716

[5] Bhatnagar S Taneja S. Zinc and cognitive development. Br J Nutr. (2001) 85:S139–45. doi: 10.1079/bjn2000306 11509102

[6] Jarosz M Olbert M Wyszogrodzka G Młyniec K Librowski T. Antioxidant and anti-inflammatory effects of zinc. Zinc-dependent Nf-κB signaling. Inflammopharmacology. (2017) 25:11–24. doi: 10.1007/s10787-017-0309-4, PMID: 28083748 PMC5306179

[7] Brenneisen P Steinbrenner H Sies H. Selenium, oxidative stress, and health aspects. Mol Asp Med. (2005) 26:256–67. doi: 10.1016/j.mam.2005.07.004, PMID: 16105679

[8] Köhrle J. Selenium and the thyroid. Curr Opin Endocrinol Diabetes Obes. (2015) 22:392–401. doi: 10.1097/MED.0000000000000190, PMID: 26313901

[9] Tarnacka B Jopowicz A Maślińska M. Copper, Iron, and manganese toxicity in neuropsychiatric conditions. Int J Mol Sci. (2021) 22:7820. doi: 10.3390/ijms22157820, PMID: 34360586 PMC8346158

[10] Haynes EN Sucharew H Kuhnell P Alden J Barnas M Wright RO. Manganese exposure and neurocognitive outcomes in rural school-age children: the communities actively researching exposure study (Ohio, USA). Environ Health Perspect. (2015) 123:1066–71. doi: 10.1289/ehp.1408993, PMID: 25902278 PMC4590758

[11] Menezes-Filho JA Bouchard M Sarcinelli Pde N Moreira JC. Manganese exposure and the neuropsychological effect on children and adolescents: a review. Rev Panam Salud Publica. (2009) 26:541–8. doi: 10.1590/s1020-49892009001200010 20107709

[12] Scheiber IF Mercer JF Dringen R. Metabolism and functions of copper in brain. Prog Neurobiol. (2014) 116:33–57. doi: 10.1016/j.pneurobio.2014.01.002, PMID: 24440710

[13] Best C Neufingerl N Van Geel L Van Den Briel T Osendarp S. The nutritional status of school-aged children: why should we care? Food Nutr Bull. (2010) 31:400–17. doi: 10.1177/156482651003100303, PMID: 20973461

[14] Jallad R Rao MS Rahman A. Prevalence of lead toxicity in adolescents in Kuwait. BMC Public Health. (2021) 21:1189. doi: 10.1186/s12889-021-11210-z, PMID: 34158008 PMC8220793

[15] Rahman A Al-Taiar A Shaban L Al-Sabah R Al-Harbi A Mojiminiyi O. Plasma 25-hydroxy vitamin D is not associated with either cognitive function or academic performance in adolescents. Nutrients. (2018) 10:1197. doi: 10.3390/nu10091197, PMID: 30200421 PMC6165454

[16] Floriańczyk B. Role of zinc in nervous system cells. J Pre-Clin Clin Res. (2011) 5:12–5.

[17] Gower-Winter SD Levenson CW. Zinc in the central nervous system: from molecules to behavior. Biofactors. (2012) 38:186–93. doi: 10.1002/biof.1012, PMID: 22473811 PMC3757551

[18] Mccall KA Huang C Fierke CA. Function and mechanism of zinc metalloenzymes. J Nutr. (2000) 130:1437s–46s. doi: 10.1093/jn/130.5.1437S, PMID: 10801957

[19] Black J Pinero D Parekh N. Zinc and cognitive development in children: perspectives from international studies. Top Clin Nutr. (2009) 24:130–8. doi: 10.1097/TIN.0b013e3181a6b947

[20] Salgueiro MJ Zubillaga MB Lysionek AE Caro RA Weill R Boccio JR. The role of zinc in the growth and development of children. Nutrition. (2002) 18:510–9. doi: 10.1016/s0899-9007(01)00812-7, PMID: 12044825

[21] Frederickson CJ Koh JY Bush AI. The neurobiology of zinc in health and disease. Nat Rev Neurosci. (2005) 6:449–62. doi: 10.1038/nrn1671, PMID: 15891778

[22] Mlyniec K. Zinc in the glutamatergic theory of depression. Curr Neuropharmacol. (2015) 13:505–13. doi: 10.2174/1570159X13666150115220617, PMID: 26412070 PMC4790399

[23] Hagmeyer S Haderspeck JC Grabrucker AM. Behavioral impairments in animal models for zinc deficiency. Front Behav Neurosci. (2014) 8:443. doi: 10.3389/fnbeh.2014.00443 25610379 PMC4285094

[24] Hambidge M. Human zinc deficiency. J Nutr. (2000) 130:1344s–9s. doi: 10.1093/jn/130.5.1344S, PMID: 10801941

[25] Chellappa A Karunanidhi S. Supplementation with iron and zinc selectively improves cognitive and behavioral functions in female adolescents. Int J Chem Eng Appl. (2012) 3:274–81. doi: 10.7763/IJCEA.2012.V3.199

[26] De Moura JE De Moura EN Alves CX Vale SH Dantas MM Silva Ade A. Oral zinc supplementation may improve cognitive function in schoolchildren. Biol Trace Elem Res. (2013) 155:23–8. doi: 10.1007/s12011-013-9766-9 23892699

[27] Kawade R. Zinc status and its association with the health of adolescents: a review of studies in India. Glob Health Action. (2012) 5:7353. doi: 10.3402/gha.v5i0.7353, PMID: 22511891 PMC3328203

[28] Sandstead HH Penland JG Alcock NW Dayal HH Chen XC Li JS. Effects of repletion with zinc and other micronutrients on neuropsychologic performance and growth of Chinese children. Am J Clin Nutr. (1998) 68:470s–5s. doi: 10.1093/ajcn/68.2.470S, PMID: 9701162

[29] Becker JS Zoriy MV Pickhardt C Palomero-Gallagher N Zilles K. Imaging of copper, zinc, and other elements in thin section of human brain samples (hippocampus) by laser ablation inductively coupled plasma mass spectrometry. Anal Chem. (2005) 77:3208–16. doi: 10.1021/ac040184q, PMID: 15889910

[30] Davies KM Hare DJ Cottam V Chen N Hilgers L Halliday G. Localization of copper and copper transporters in the human brain. Metallomics. (2013) 5:43–51. doi: 10.1039/c2mt20151h, PMID: 23076575

[31] Lutsenko S Bhattacharjee A Hubbard AL. Copper handling machinery of the brain. Metallomics. (2010) 2:596–608. doi: 10.1039/c0mt00006j, PMID: 21072351

[32] Opazo CM Greenough MA Bush AI. Copper: from neurotransmission to neuroproteostasis. Front Aging Neurosci. (2014) 6:143. doi: 10.3389/fnagi.2014.00143, PMID: 25071552 PMC4080678

[33] Bortolato M Shih JC. Behavioral outcomes of monoamine oxidase deficiency: preclinical and clinical evidence. Int Rev Neurobiol. (2011) 100:13–42. doi: 10.1016/B978-0-12-386467-3.00002-9, PMID: 21971001 PMC3371272

[34] Prohaska JR. Impact of copper deficiency in humans. Ann N Y Acad Sci. (2014) 1314:1–5. doi: 10.1111/nyas.12354, PMID: 24517364

[35] Beliaev A Ferreira H Learmonth AD Soares-Da-Silva P. Dopamine β-monooxygenase: mechanism, substrates and inhibitors. Curr Enzym Inhib. (2009) 5:27–43. doi: 10.2174/157340809787314265

[36] Bousquet-Moore D Mains RE Eipper BA. Peptidylgycine α-amidating monooxygenase and copper: a gene-nutrient interaction critical to nervous system function. J Neurosci Res. (2010) 88:2535–45. doi: 10.1002/jnr.22404, PMID: 20648645 PMC3732055

[37] Klevay LM. Alzheimer's disease as copper deficiency. Med Hypotheses. (2008) 70:802–7. doi: 10.1016/j.mehy.2007.04.051, PMID: 17928161

[38] Salustri C Barbati G Ghidoni R Quintiliani L Ciappina S Binetti G. Is cognitive function linked to serum free copper levels? A cohort study in a normal population. Clin Neurophysiol. (2010) 121:502–7. doi: 10.1016/j.clinph.2009.11.090, PMID: 20097602

[39] Zhou G Ji X Cui N Cao S Liu C Liu J. Association between serum copper status and working memory in schoolchildren. Nutrients. (2015) 7:7185–96. doi: 10.3390/nu7095331, PMID: 26343713 PMC4586526

[40] Prohaska JR. Functions of trace elements in brain metabolism. Physiol Rev. (1987) 67:858–901. doi: 10.1152/physrev.1987.67.3.858, PMID: 3299411

[41] Bowman AB Kwakye GF Herrero Hernández E Aschner M. Role of manganese in neurodegenerative diseases. J Trace Elem Med Biol. (2011) 25:191–203. doi: 10.1016/j.jtemb.2011.08.144, PMID: 21963226 PMC3230726

[42] Chen P Chakraborty S Mukhopadhyay S Lee E Paoliello MM Bowman AB. Manganese homeostasis in the nervous system. J Neurochem. (2015) 134:601–10. doi: 10.1111/jnc.13170, PMID: 25982296 PMC4516557

[43] Wedler FC Denman RB. Glutamine synthetase: the major Mn(II) enzyme in mammalian brain. Curr Top Cell Regul. (1984) 24:153–69. doi: 10.1016/b978-0-12-152824-9.50021-6, PMID: 6149889

[44] Horning KJ Caito SW Tipps KG Bowman AB Aschner M. Manganese is essential for neuronal health. Annu Rev Nutr. (2015) 35:71–108. doi: 10.1146/annurev-nutr-071714-034419, PMID: 25974698 PMC6525788

[45] Zachara BA Pawluk H Bloch-Boguslawska E Sliwka KM Korenkiewicz J Skok Z. Tissue level, distribution, and total body selenium content in healthy and diseased humans in Poland. Arch Environ Health. (2001) 56:461–6. doi: 10.1080/00039890109604483, PMID: 11777029

[46] Whanger PD. Selenium and the brain: a review. Nutr Neurosci. (2001) 4:81–97. doi: 10.1080/1028415X.2001.11747353, PMID: 11842884

[47] Solovyev ND. Importance of selenium and selenoprotein for brain function: from antioxidant protection to neuronal signalling. J Inorg Biochem. (2015) 153:1–12. doi: 10.1016/j.jinorgbio.2015.09.003, PMID: 26398431

[48] Cobley JN Fiorello ML Bailey DM. 13 reasons why the brain is susceptible to oxidative stress. Redox Biol. (2018) 15:490–503. doi: 10.1016/j.redox.2018.01.008, PMID: 29413961 PMC5881419

[49] Cardoso BR Roberts BR Bush AI Hare DJ. Selenium, selenoproteins and neurodegenerative diseases. Metallomics. (2015) 7:1213–28. doi: 10.1039/c5mt00075k, PMID: 25996565

[50] Reeves MA Hoffmann PR. The human selenoproteome: recent insights into functions and regulation. Cell Mol Life Sci. (2009) 66:2457–78. doi: 10.1007/s00018-009-0032-4, PMID: 19399585 PMC2866081

[51] Schweizer U Dehina N Schomburg L. Disorders of selenium metabolism and selenoprotein function. Curr Opin Pediatr. (2011) 23:429–35. doi: 10.1097/MOP.0b013e32834877da, PMID: 21670677

[52] Samuels MH. Psychiatric and cognitive manifestations of hypothyroidism. Curr Opin Endocrinol Diabetes Obes. (2014) 21:377–83. doi: 10.1097/MED.0000000000000089, PMID: 25122491 PMC4264616

[53] Tarım Ö. Thyroid hormones and growth in health and disease. J Clin Res Pediatr Endocrinol. (2011) 3:51–5. doi: 10.4274/jcrpe.v3i2.11, PMID: 21750631 PMC3119440

[54] Amorós R Murcia M Ballester F Broberg K Iñiguez C Rebagliato M. Selenium status during pregnancy: influential factors and effects on neuropsychological development among Spanish infants. Sci Total Environ. (2018) 610-611:741–9. doi: 10.1016/j.scitotenv.2017.08.042, PMID: 28822941

[55] Amorós R Murcia M González L Rebagliato M Iñiguez C Lopez-Espinosa MJ. Maternal selenium status and neuropsychological development in Spanish preschool children. Environ Res. (2018) 166:215–22. doi: 10.1016/j.envres.2018.06.002, PMID: 29890426

[56] Berr C Arnaud J Akbaraly TN. Selenium and cognitive impairment: a brief-review based on results from the Eva study. Biofactors. (2012) 38:139–44. doi: 10.1002/biof.1003, PMID: 22419540

[57] Gao S Jin Y Hall KS Liang C Unverzagt FW Ji R. Selenium level and cognitive function in rural elderly Chinese. Am J Epidemiol. (2007) 165:955–65. doi: 10.1093/aje/kwk073, PMID: 17272290 PMC2760949

[58] Skröder H Kippler M Tofail F Vahter M. Early-life selenium status and cognitive function at 5 and 10 years of age in Bangladeshi children. Environ Health Perspect. (2017) 125:117003. doi: 10.1289/EHP1691, PMID: 29116931 PMC5947942

[59] Pasco JA Jacka FN Williams LJ Evans-Cleverdon M Brennan SL Kotowicz MA. Dietary selenium and major depression: a nested case-control study. Complement Ther Med. (2012) 20:119–23. doi: 10.1016/j.ctim.2011.12.008, PMID: 22500660

[60] Ellwanger JH Franke SI Bordin DL Prá D Henriques JA. Biological functions of selenium and its potential influence on Parkinson's disease. An Acad Bras Cienc. (2016) 88:1655–74. doi: 10.1590/0001-3765201620150595, PMID: 27556332

[61] Schweizer U Bräuer AU Köhrle J Nitsch R Savaskan NE. Selenium and brain function: a poorly recognized liaison. Brain Res Brain Res Rev. (2004) 45:164–78. doi: 10.1016/j.brainresrev.2004.03.004, PMID: 15210302

[62] Hess SY. National risk of zinc deficiency as estimated by national surveys. Food Nutr Bull. (2017) 38:3–17. doi: 10.1177/0379572116689000, PMID: 28118744

[63] Wessells KR Brown KH. Estimating the global prevalence of zinc deficiency: results based on zinc availability in national food supplies and the prevalence of stunting. PLoS One. (2012) 7:e50568. doi: 10.1371/journal.pone.0050568, PMID: 23209782 PMC3510072

[64] Abeywickrama HM Koyama Y Uchiyama M Shimizu U Iwasa Y Yamada E. Micronutrient status in Sri Lanka: a review. Nutrients. (2018) 10:1583. doi: 10.3390/nu10111583, PMID: 30373264 PMC6265675

[65] Ministry of Public Health, Afghanistan, and UNICEF Afghanistan National Nutrition Survey 2013. (2014).

[66] Morales-Ruán Mdel C Villalpando S García-Guerra A Shamah-Levy T Robledo-Pérez R Avila-Arcos MA. Iron, zinc, copper and magnesium nutritional status in Mexican children aged 1 to 11 years. Salud Publica Mex. (2012) 54:125–34. doi: 10.1590/s0036-36342012000200008 22535171

[67] Rahman S Ahmed T Rahman AS Alam N Ahmed AM Ireen S. Status of zinc nutrition in Bangladesh: the underlying associations. J Nutr Sci. (2016) 5:e25. doi: 10.1017/jns.2016.17, PMID: 27547388 PMC4976114

[68] Kenya National Bureau of Statistics, Kenya Medical Research Institute, Division of Nutrition, Ministry of Public Health and Sanitation. Kenya National Micronutrient Survey 2011. (2011).

[69] Ministry of National Health Services, Regulations & Coordination (Pakistan), United Nations Children’s Fund (UNICEF), Aga Khan University, Pakistan Medical Research Council, & UK Aid Direct. National Nutrition Survey 2018. [Pakistan]. (2018).

[70] Abdelrahim I Mahgoub HM Mohamed AA Ali NI Elbashir MI Adam I. Anaemia, folate, zinc and copper deficiencies among adolescent schoolgirls in eastern Sudan. Biol Trace Elem Res. (2009) 132:60–6. doi: 10.1007/s12011-009-8397-7, PMID: 19430735

[71] Dabbaghmanesh MH Salehi NM Siadatan J Omrani GR. Copper concentration in a healthy urban adult population of southern Iran. Biol Trace Elem Res. (2011) 144:217–24. doi: 10.1007/s12011-011-9074-1, PMID: 21573871

[72] Olivares M Lera L Albala C Pizarro F Araya M. Prevalence of zinc and copper deficiencies in older subjects living in metropolitan Santiago. Rev Med Chile. (2011) 139:283–9. doi: 10.4067/S0034-98872011000300001, PMID: 21879158

[73] Abiaka C Olusi S Al-Awadhi A. Reference ranges of copper and zinc and the prevalence of their deficiencies in an Arab population aged 15-80 years. Biol Trace Elem Res. (2003) 91:33–43. doi: 10.1385/BTER:91:1:33, PMID: 12713027

[74] Buxaderas SC Farré-Rovira R. Whole blood and serum zinc levels in relation to sex and age. Rev Esp Fisiol. (1985) 41:463–70. 4095368

[75] Buxaderas SC Farré-Rovira R. Whole blood and serum copper levels in relation to sex and age. Rev Esp Fisiol. (1986) 42:213–7. 3749578

[76] Zhang H Cao Y Man Q Li Y Lu J Yang L. Study on reference range of zinc, copper and copper/zinc ratio in childbearing women of China. Nutrients. (2021) 13:946. doi: 10.3390/nu13030946, PMID: 33804217 PMC7999022

