# Supplementary material for: Blood levels of copper, manganese, selenium, and zinc are positively associated with cognitive function and academic performance in adolescents
Source: Front Nutr. 2025 Jul 24;12:1638283. doi: 10.3389/fnut.2025.1638283 (PMC12330213; doi:10.3389/fnut.2025.1638283)
Supplement: Supplementary file 1 [file Table_1.docx]

SupplementaryTable 1: Comparisons of Blood levels (µg/dL) of Cu, Mn, Se and Zn across selected studies:

| **Country/Study** | **Subjects** | **Parameter** | **Cu** | **Mn** | **Se** | **Zn** |
| --- | --- | --- | --- | --- | --- | --- |
| Kuwait/This study | Children  11-14 years | Median  (5^th^ -95^th^) * | 22.5  (10.4-48.8) | 6.03  (1.35-20.77) | 34.2  (12.7-82.3) | 164  (80-337) |
| Congo/Tuakuila et al. 2014; PMID: 24210171. | Children  <6 years | 5^th^ -95^th^ * | 90-170 | 0.57-2.22 | 8.5-13.6 | 340-690 |
| Sweden/Bárány et al. 2002; PMID: 12051788. | Children  15 years | Median  (2.5^th^ - 9.5^th^) * | 92  (73-130) |  | 11  (6.5-16.0) | 610  (450-810) |
| Australia/Komarova et al. 2021; PMID: 33800753. | Adults  18-73 years | Mean  (range) | 85  (65-142) | 0.98  (0.45-1.95 | 14.1  (11.8-22.4 | 672  (462-925) |
| Benin/Yedomon et al. 2017; PMID: 27847219. | Adults  18-65 y | GM  (5th-95th) | 87  (72-103) | 1.97  (1.57-2.51) | 16.3  (12.3-20.5) | 485  (368-667) |
| Germany/Heitland et al. 2006; PMID: 17098585. | Adults | GM  (5th-95th) | 102  (80-162) | 0.86  (0.57-1.46) | 13.2  (10.5-16.4) |  |
| Italy/Bocca et al. 2011; PMID: 21242073. | Men | GM  (5th-95th) | 96  (77-120.) | 0.80  (0.46-1.42) | 14.1  (11.1-18.5) | 680  (506-896) |
| Norway/Syversen et al. 2021; PMID: 32897510. | Adults  49-66 y | Median  (10^th^ -95^th^) * | 101  (87-137) | 0.96  (0.61-1.38) | 11.3  (9.1-14.5) | 760  (630-1080) |
| Norway/Simić et al. 2022; PMID: 34634345. | Adults  20-91 y | GM  (5^th^ – 95^th^) * | 101  (82-127) | 0.91  (0.58-1.49) | 10.0  (7.5-13.7) | 750  (590-910) |
| Sweden/Schultze et al. 2014; PMID: 24329009. | Adults  70 y | Median  (IQR) | 82  (74-90) | 0.75  (0.63-0.91) |  | 628  (573-680) |
| Slovenia/Snoj Tratnik et al. 2019; PMID: 30878540. | Adults  18-49 y | GM  (5^th^ – 95^th^) * | 95  (74-126) | 1.38  (0.82-2.62) | 10.5  (7.4-15.2) | 660  (520-830) |
| Canada/Saravanabhavan et al. 2017; PMID: 27776932. | Adults  3-79 y | Ref Values  at 95^th^ * | 100 (m)  130 (f) | 1.6 (6-19 years) | 22 (6-19 years) | 650 (m);  680 (f)  6-19 years |
| China/Zhang et al. 2015; PMID: 25836720. | Adults  17-60 Y | GM  (25^th^ – 95^th^) * | 80  (69-89) | 1.14  (0.88-1.47) |  | 467  (378-556) |
| China/Zhang et al. 2021; PMID: 33804217. | Women  18-44 y | GM  (2.5^th^ - 97.5^th^) * | 93  (92-125) |  |  | 539  (403-738) |

*, these are percentiles.
